# The Role of Mitochondrial DNA in Modulating Chemoresistance in Esophageal Cancer: Mechanistic Insights and Therapeutic Potential

**DOI:** 10.3390/biom15081128

**Published:** 2025-08-05

**Authors:** Koji Tanaka, Yasunori Masuike, Yuto Kubo, Takashi Harino, Yukinori Kurokawa, Hidetoshi Eguchi, Yuichiro Doki

**Affiliations:** 1 Departments of Gastroenterological Surgery, Graduate School of Medicine, Osaka University, Suita 565-0871, Japan; 2 Department of Surgery, Osaka International Cancer Institute, Osaka 541-8567, Japan; 3 Department of Esophageal Surgery, National Cancer Center Hospital East, Kashiwa 277-8577, Japan; 4 Department of Surgery, Kansai Medical University, Hirakata 573-1191, Japan

**Keywords:** mitochondrial DNA, esophageal cancer, chemotherapy resistance, mtDNA mutations, oxidative phosphorylation, mitochondrial-targeted therapy

## Abstract

Chemotherapy remains a cornerstone in the treatment of esophageal cancer (EC), yet chemoresistance remains a critical challenge, leading to poor outcomes and limited therapeutic success. Mitochondrial DNA (mtDNA) has emerged as a pivotal player in mediating these responses, influencing cellular metabolism, oxidative stress regulation, and apoptotic pathways. This review provides a comprehensive overview of the mechanisms by which mtDNA alterations, including mutations and copy number variations, drive chemoresistance in EC. Specific focus is given to the role of mtDNA in metabolic reprogramming, including its contribution to the Warburg effect and lipid metabolism, as well as its impact on epithelial–mesenchymal transition (EMT) and mitochondrial bioenergetics. Recent advances in targeting mitochondrial pathways through novel therapeutic agents, such as metformin and mitoquinone, and innovative approaches like CRISPR/Cas9 gene editing, are also discussed. These interventions highlight the potential for overcoming chemoresistance and improving patient outcomes. By integrating mitochondrial diagnostics with personalized treatment strategies, we propose a roadmap for future research that bridges basic mitochondrial biology with translational applications in oncology. The insights offered in this review emphasize the critical need for continued exploration of mtDNA-targeted therapies to address the unmet needs in EC management and other diseases associated with mitochondria.

## 1. Introduction

Esophageal cancer (EC) is a leading cause of cancer-related mortality, ranking as the eighth most common malignancy globally [1,2,3,4]. It is characterized by its aggressive nature, late-stage diagnosis, and poor overall prognosis, with a five-year survival rate below 20% for advanced cases [5,6,7]. While multimodal treatment strategies, including surgery, radiation, and chemotherapy, have improved outcomes, the clinical management of advanced EC remains challenging [8]. Chemoresistance, defined as the failure to respond to chemotherapeutic regimens, represents one of the most significant barriers to effective treatment and contributes to disease progression and recurrence. Recent epidemiological evidence has shown that deficiencies in micronutrients such as selenium, zinc, and vitamin E, as well as high dietary exposure to acetaldehyde from alcohol consumption and nitrosamines from smoked foods, are associated with an increased risk of esophageal squamous cell carcinoma (ESCC) [5,9,10,11]. These dietary risk factors may contribute to mitochondrial damage and oxidative stress, promoting carcinogenesis and chemoresistance [12].

**Figure 1:**
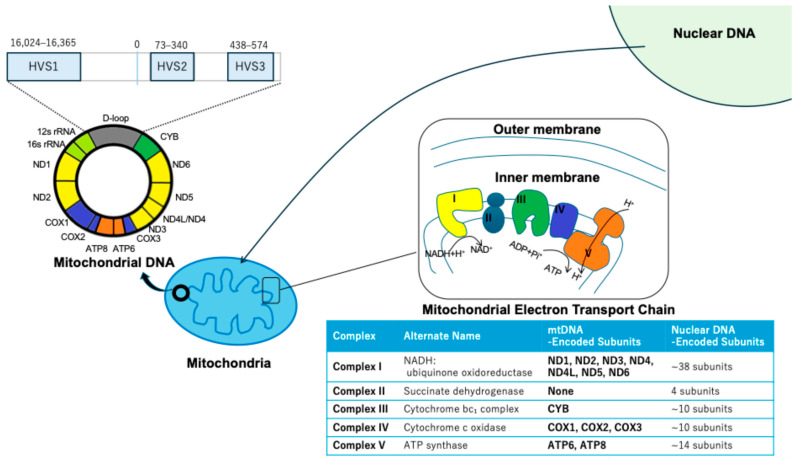
Schematic of human mtDNA genome. A graphical representation of the human mitochondrial DNA (mtDNA) genome, highlighting the displacement loop (D-loop) region and hypervariable segments (HVS1, HVS2, and HVS3). The positions of nuclear DNA and mitochondrial structures are also indicated.

Human mtDNA is a 16,569 bp circular double-stranded molecule, encoding 13 essential subunits of complexes I, III, IV, and V of the electron transport chain (ETC), which are synthesized by mitochondrial ribosomes using mtDNA-encoded transcripts, independent of nuclear transcription, alongside 22 tRNAs and 2 rRNAs for intra-mitochondrial translation. Complex II is the only ETC complex fully encoded by nuclear DNA (Figure 1). Mitochondria import over 99% of mitochondrial proteins. These proteins are encoded by nuclear DNA, synthesized in the cytosol, and imported via the TOM/TIM complex into the mitochondrial matrix or inner membrane. The D-loop region of mitochondrial DNA contains hypervariable segments (HVS), particularly HVS-I and HVS-II, which exhibit high mutation rates. These regions are frequently analyzed in population genetics, forensic studies, and phylogenetics due to their variability and maternal inheritance, providing valuable insights into human migration and lineage tracing. The copy number is tightly regulated (100–1000 copies per cell) by nuclear-encoded factors: TFAM packages nucleoids, POLG conducts replication, TFB2M initiates transcription, and PGC-1α orchestrates mitochondrial biogenesis [13]. Unlike nuclear DNA, mtDNA is maternally inherited and exists in multiple copies per cell, rendering it vulnerable to mutations and copy number variations [14,15]. Mitochondria remain crucial for regulating oxidative stress, cell survival, and drug-induced apoptosis. Unlike nuclear DNA, mtDNA is highly susceptible to mutations and copy number variations due to its limited repair mechanisms and proximity to reactive oxygen species (ROS) generated during oxidative phosphorylation (OXPHOS) [16,17]. Common mtDNA lesions include 8-oxo-7,8-dihydroguanine (8-oxoG)**,** abasic (AP) sites, and single-strand breaks, all of which can compromise transcription and replication fidelity [18]. Persistent mtDNA damage may lead to mutations that impair ETC complex assembly and function, ultimately contributing to mitochondrial dysfunction and reduced responsiveness to chemotherapeutic agents. Preserving mtDNA integrity is essential for mitochondrial function, as mtDNA encodes key subunits of the oxidative phosphorylation complexes. Despite its compact nature and limited DNA repair capacity compared to the nuclear genome, mitochondria harbor several dedicated repair pathways that maintain genome stability under metabolic stress [19]. Among these, base excision repair (BER) is the most prominent and well-characterized pathway in mitochondria, responsible for repairing oxidized bases, abasic sites, and single-strand breaks. Key mitochondrial BER enzymes include OGG1, NEIL1, APE1, DNA polymerase γ, and DNA ligase III [20]. In addition, mitochondria may exhibit mismatch repair (MMR)-like activity, although it lacks canonical MMR proteins found in the nucleus [21]. Dysregulation or deficiency in these repair systems contributes to various pathologies, including neurodegenerative diseases, aging, and cancer [22].

Mutations in mtDNA can disrupt the normal function of mitochondria, particularly affecting the electron transport chain and oxidative phosphorylation. This dysfunction can lead to the following: (1) changes in the production of reactive oxygen species (ROS), (2) changes in energy production pathways, and (3) altered apoptotic signaling. These changes can collectively contribute to chemoresistance by allowing cancer cells to survive under conditions that would normally trigger cell death.

Furthermore, mtDNA exists in a dynamic state within cells, undergoing continuous replication and degradation to maintain mitochondrial function [23]. Quality control hinges on mitophagy mediated by PINK1/Parkin and proteostasis networks that eliminate damaged mtDNA or mitochondria. This dynamic regulation, coupled with the presence of multiple mtDNA copies per cell, allows cancer cells to manipulate their mitochondrial content in response to environmental stresses [24], including chemotherapy. The disruption of these processes can have profound effects on cellular homeostasis and therapeutic outcomes.

This review aims to provide a comprehensive overview of the role of mtDNA in chemoresistance in EC. We explore the structural and functional characteristics of mtDNA, its alterations in EC, and their mechanistic links to drug resistance. Additionally, we discuss emerging therapeutic approaches that target mitochondrial pathways, highlighting their potential to improve treatment outcomes. By bridging basic mitochondrial biology with translational research, this review seeks to identify novel avenues for overcoming chemoresistance and advancing the clinical management of EC.

## 2. Clinical Correlations of mtDNA Alterations in EC

### 2.1. Prognostic Impact of mtDNA Copy Number Variations

Masuike et al. measured mtDNA copy numbers via real-time PCR in 80 resected ESCC specimens (adjusted relative to the TE11 cell line as 1.00) [25]. The median mtDNA copy number of tumor tissues was median 0.56 (0.30 ± 1.47), whereas paired noncancerous mucosa was median 0.95 (0.58 ± 1.40). The median mtDNA copy number of ESCC samples from 80 patients was 0.91 (0.09 ± 1.98). Tumors with low mtDNA (<0.91 relative units) exhibited a median overall survival (OS) of 14.2 months versus 28.7 months in tumors with high mtDNA (hazard ratio (HR) = 2.31; 95% confidence interval (CI): 1.18–4.50; *p* = 0.015), as well as significantly shorter recurrence-free survival (RFS) (9.6 vs. 21.4 months; HR = 2.45; 95% CI: 1.28–4.68; *p* = 0.009) after adjusting for clinico-pathologic variables. Furthermore, a low mtDNA copy number correlated with advanced pathological T stage (*p* = 0.045) and overall stage (*p* = 0.025), suggesting a role in ESCC progression. On multivariate Cox regression, a low mtDNA copy number emerged as an independent predictor of poor OS (HR 2.281; 95% CI 1.144–4.781; *p* = 0.019), along with the pathological T stage (HR 2.301; 95% CI 1.116–4.891; *p* = 0.024). These findings underscore that mtDNA depletion confers aggressive tumor behavior and confers a survival disadvantage in EC patients.

### 2.2. D-Loop HVS1 Mutations Correlate with Chemotherapy Response

The D-loop region of mtDNA spans 1122–1552 bp and contains hypervariable segments (HVS1: 16,024–16,383 bp; HVS2: 52–372 bp; HVS3: 438–574 bp), which are mutation hotspots due to their single-stranded conformation and high replication frequency. Challen et al. conducted a seminal investigation into the clinical relevance of mitochondrial DNA (mtDNA) mutations in head and neck squamous cell carcinoma (HNSCC) [26]. This study systematically analyzed 6 oral carcinoma cell lines and 34 paired tumor-normal tissue samples through full mitochondrial genome screening (∼100 kb coverage using 114 amplicons), employing denaturing high-performance liquid chromatography (DHPLC) for mutation detection. The research specifically targeted hypervariable segment 1 (HVS1) within the D-loop region, a critical regulatory area for mtDNA replication. The analysis revealed mtDNA mutations in 50% of cell lines (3/6) and 18% of tumor tissues (6/34). There was no correlation between D-loop mutations and determinates of clinical outcome; specifically, the tumor stage and the expression of hypoxia-inducible genes included in a highly prognostic hypoxia metagene. The authors concluded that mtDNA D-loop mutations in HNSCC likely represent bystander effects of genomic instability rather than functional drivers.

**Figure 2:**
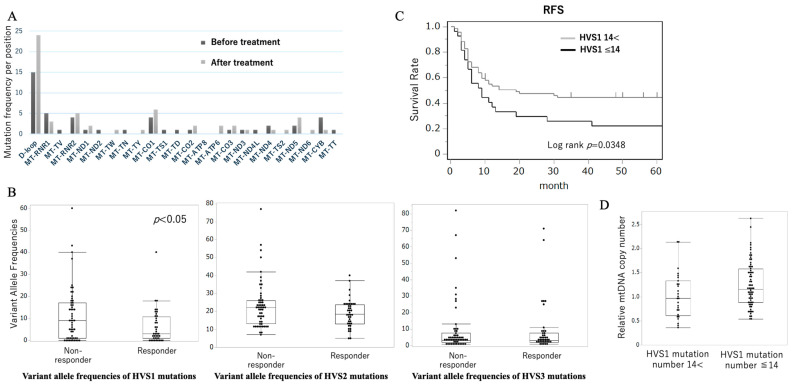
Variant Allele Frequencies of mtDNA Mutations in ESCC After Chemotherapy. (**A**) Comparison of mtDNA mutation frequencies before and after chemotherapy. (**B**) Comparison of Variant Allele Frequencies of HVS1,2, and 3 mutations between a responder and non-responder. (**C**) Kaplan–Meier survival curves comparing overall survival in patients with HVS1 mutation frequencies ≤ 14 versus > 14 (Log-rank *p* = 0.0348). (**D**) Comparison of the mtDNA copy number between the HVS1 mutations high and low group. Figure adapted from Harino et al. (2024) [27].

On the other hand, Harino et al. reported on the role of mitochondrial DNA (mtDNA) D-loop mutations in chemotherapy resistance among ESCC patients. Sequencing (average coverage > 5000×) of the D-loop region in 27 ESCC patients pre- and post-cisplatin therapy was performed [27]. As shown in Figure 2A, the frequency of mutations per nucleotide position was markedly higher in the D-loop region compared to other mitochondrial loci. In a larger cohort of 96 ESCC patients undergoing neoadjuvant chemotherapy, targeted resequencing of the D-loop region revealed that HVS1 mutations were the strongest predictor of chemotherapeutic resistance [27]. A receiver operating characteristic (ROC) analysis yielded an area under the curve (AUC) of 0.631 for HVS1 mutation count and identified ≥ 14 mutations as the optimal cutoff for predicting resistance. Patients with ≥14 HVS1 mutations exhibited significantly higher rates of pathological nonresponse (PR/CR rate: 28% vs. 72%, *p* = 0.002, Figure 2B) and shortened recurrence-free survival (5-year RFS= 22% vs. 56%, *p* = 0.035, Figure 2C), confirming that the D-loop mutational burden is clinically relevant. Moreover, HVS1-mutant tumors had a significantly lower mtDNA copy number compared to those with <14 HVS1 mutations (*p* = 0.0242), suggesting that D-loop alterations may compromise mtDNA replication and maintenance, thereby promoting chemoresistance. These variants are often mapped to transcription factor-binding sites, suggesting functional impact on mtDNA replication. This work provides the first evidence linking chemotherapy-induced mtDNA mutations to acquired resistance in ESCC, offering mechanistic insights into treatment failure. These results are constrained by the results by Challen et al. Differences in cancer types (HNSCC vs. ESCC), sequencing depth, and cohort size may account for the divergent findings, and functional or larger studies are required to validate these findings.

## 3. Mechanistic Pathways Linking mtDNA Dysfunction to Chemoresistance

MtDNA alterations promote chemoresistance through interconnected pathways that reprogram cellular metabolism, modulate redox homeostasis, enforce EMT/CSC phenotypes, alter mitochondrial membrane potential, and engender epigenetic changes. Below, we detail these mechanisms, integrating evidence from both in vitro models and clinical specimens.

### 3.1. Bioenergetic Reprogramming and Metabolic Adaptation

Loss or mutation of mtDNA-encoded ETC subunits significantly diminishes OXPHOS capacity. Masuike et al. demonstrated that the knockdown of mitochondrial transcription factor A (TFAM) in human ESCC cell lines TE8 and TE11 significantly reduced the mitochondrial DNA (mtDNA) copy number. This decrease in mtDNA led to impaired mitochondrial function, evidenced by mitochondrial swelling, disrupted cristae structure, and enhanced lactate production, indicating a metabolic shift toward glycolysis. Under normoxic conditions, these mtDNA-depleted cells exhibited reduced proliferation rates compared to controls; however, they maintained robust proliferation under hypoxic conditions, reflecting a compensatory glycolytic adaptation [25]. Similar bioenergetic adaptations—characterized by reduced oxidative phosphorylation capacity and elevated glycolysis—have been reported across multiple cancer models of mtDNA depletion, highlighting the central role of mitochondrial dysfunction in metabolic reprogramming [28]. Mitochondrial dysfunction drives metabolic reprogramming in cancer, often characterized by increased glycolytic flux and the diversion of metabolites into biosynthetic pathways. Pyruvate kinase M2 (PKM2) plays a central role in this adaptation by regulating the balance between glycolysis and the pentose phosphate pathway (PPP), thus supporting NADPH production and redox homeostasis. In ESCC, PKM2 has been shown to modulate chemotherapy sensitivity through PPP activation, highlighting its potential as a metabolic target [29]. This “Warburg-like” adaptation supports ATP production and biosynthesis under chemotherapeutic stress.

### 3.2. ROS Signaling and Redox Homeostasis

Reactive oxygen species (ROS), while often considered harmful metabolic byproducts, play paradoxical roles in cancer development, progression, and therapy response. Among the major intracellular sources of ROS, mitochondria generate superoxide (O_2_•^−^) through electron leakage from complexes I and III of the electron transport chain (ETC), especially under stress or mitochondrial dysfunction [30]. This superoxide is rapidly converted by mitochondrial manganese superoxide dismutase (MnSOD/SOD2) into hydrogen peroxide (H_2_O_2_), which, due to its membrane permeability, becomes a central mediator of redox signaling. In esophageal epithelial cells, mitochondrial SOD2 (MnSOD) has been shown to play a critical role in regulating redox balance during epithelial–mesenchymal transition (EMT) and in the dynamic conversion of cellular phenotypes defined by differential CD44 expression. Kinugasa et al. demonstrated that TGF-β–induced EMT leads to a phenotypic shift from CD44^low/CD24^high (CD44L) epithelial cells to CD44^high/CD24^low (CD44H) mesenchymal cells, a transition that is tightly coupled to SOD2 induction [31]. Notably, SOD2 upregulation in CD44H cells was mediated by the transcription factors NF-κB and ZEB2, and served to suppress intracellular ROS levels, thus promoting the survival and stability of mesenchymal-like populations. Functionally, SOD2 knockdown enhanced ROS accumulation and increased sensitivity to oxidative stress in CD44L cells, accelerating their transition to the CD44H phenotype upon TGF-β stimulation. This suggests that SOD2-mediated antioxidant capacity acts as a rheostat, fine-tuning ROS to levels that permit EMT without triggering cell death or senescence. Intriguingly, the study also found that a short (1.5 kb) transcript variant of SOD2, less susceptible to microRNA-mediated suppression, was preferentially expressed in CD44H cells, highlighting an additional post-transcriptional layer of regulation. These findings support the concept that SOD2 is not merely a passive antioxidant enzyme, but rather a dynamic modulator of cell state transitions, including those associated with stem-like properties and drug resistance. In the context of esophageal cancer, where CD44H-like subpopulations have been linked to tumor initiation, invasion, and therapy resistance, the SOD2–ROS axis may serve as a crucial determinant of phenotypic plasticity and treatment outcomes.

NRF2 is a redox-sensitive transcription factor that becomes stabilized and activated in response to oxidative stress. Under basal conditions, the NRF2 is bound to the KEAP1 in the cytoplasm and targeted for proteasomal degradation. The oxidation of cysteine residues in the KEAP1 upon ROS accumulation disrupts this interaction, allowing the NRF2 to translocate into the nucleus [32]. Once in the nucleus, the NRF2 binds to antioxidant response elements (AREs) and activates the transcription of a broad array of antioxidant and cytoprotective genes, such as: GPX1 (glutathione peroxidase 1), PRDX3 (peroxiredoxin 3), NQO1, and HO-1 (heme oxygenase 1). This gene expression program helps maintain redox homeostasis and provides resistance to chemotherapeutic agents, particularly those that rely on ROS generation (e.g., anthracyclines, platinum compounds). The NRF2-KEAP1 pathway is aberrantly activated in many ESCC tumors. Somatic mutations in the NFE2L2 (encoding NRF2) or the KEAP1 are detected in up to 10–20% of ESCCs, leading to constitutive NRF2 activation [33].

A pivotal study by Wang et al. demonstrated that the NRF2 directly regulates the transcription of several ATP-binding cassette (ABC) transporter genes, including ABCC1 (MRP1), ABCC2 (MRP2), and ABCG2 (BCRP) [34]. These transporters function as drug efflux pumps that reduce intracellular concentrations of chemotherapeutic agents, thereby diminishing their cytotoxic effects. Mechanistically, Wang et al. showed that NRF2 binds to antioxidant response elements (AREs) located in the promoters of these genes and enhances their transcriptional activity, as confirmed by promoter-reporter assays and chromatin immunoprecipitation (ChIP) experiments. Overexpression of the NRF2 in human cancer cell lines led to a marked increase in mRNA and protein levels of ABCC1, ABCC2, and ABCG2, whereas silencing NRF2 by siRNA significantly reduced their expression. Functionally, NRF2 overactivation rendered cancer cells more resistant to multiple chemotherapeutic drugs, including doxorubicin, etoposide, and cisplatin, while NRF2 knockdown restored drug sensitivity. These findings underscore the role of NRF2 as a mediator of drug resistance via the transcriptional activation of ABC transporters in several malignancies. Although direct evidence linking the NRF2-mediated transcriptional activation of ABC transporters to chemoresistance in esophageal cancer (EC) is currently limited, the overexpression of ABC transporters in ESCC, combined with frequent NRF2 pathway alterations, suggests a potential mechanistic link [34].

ROS also regulate the stability of hypoxia-inducible factor-1α (HIF-1α), which under normoxic conditions is hydroxylated by prolyl hydroxylases (PHDs), leading to its degradation. However, H_2_O_2_ can inhibit PHD activity, leading to HIF-1α stabilization even in normoxia [35]. Stabilized HIF-1α transactivates the following: glycolytic enzymes (HK2, PFK1, LDHA), PDK1, and VEGF [36]. This metabolic shift to aerobic glycolysis (Warburg effect) not only limits further mitochondrial ROS production but also supports survival under chemotherapeutic or hypoxic stress.

Additionally, the ROS-mediated activation of ATM/ATR checkpoints enhances DNA repair capacity, promoting tolerance to DNA crosslinks induced by platinum drugs [37]. Thus, mtDNA alteration-induced ROS overproduction constitutes a double-edged sword: low to moderate levels promote survival via redox adaptation and DNA repair, while excessive ROS can trigger apoptosis [38]. Cancer cells often exploit this balance to their advantage, reinforcing chemoresistance.

In addition to apoptosis, ferroptosis—a regulated cell death driven by iron accumulation and lipid peroxidation—has emerged as a critical pathway. In ESCC models, suppression of glutathione peroxidase 4 (GPX4) and overexpression of ACSL4 have been linked to enhanced sensitivity to ferroptosis-inducing agents [39,40]. Targeting ferroptosis regulators may offer a novel strategy to overcome mitochondrial dysfunction-driven chemoresistance.

### 3.3. Epithelial–Mesenchymal Transition and Cancer Stem Cell Phenotypes

The EMT is a dynamic transdifferentiation program whereby epithelial cells lose polarity and cell–cell adhesion (marked by the downregulation of E-cadherin) and acquire mesenchymal traits (the upregulation of N-cadherin, vimentin, and transcription factors such as ZEB1, Snail, and Twist) [41]. EMT is implicated in enhanced migratory/invasive capacity, a resistance to apoptosis, and generation of CSC subpopulations—key drivers of chemoresistance and tumor relapse [42].

Masuike et al. demonstrated that the depletion of mtDNA in TE11 cells through TFAM knockdown induced a pronounced shift toward a mesenchymal phenotype. Treated cells adopted a spindle-shaped, fibroblast-like morphology, accompanied by a marked reduction in epithelial marker expression; E-cadherin protein levels decreased significantly as quantified by Western blot. Concurrently, mesenchymal markers were substantially upregulated, with N-cadherin and vimentin expression both increasing, as measured by quantitative PCR. These molecular changes were orchestrated by the elevated expression of key EMT transcription factors such as ZEB1, which was found to be significantly higher in mtDNA-depleted cells. Increased migratory and invasive behaviors in Matrigel invasion assays and scratch-wound assays was observed [25].

Flow cytometric analysis further revealed enrichment of CD44+ cancer stem cell-like populations in TFAM-silenced TE11 cells, and sphere formation assays confirmed increased self-renewal capacity. In functional cytotoxicity assays, mtDNA-depleted cells exhibited significantly higher survival rates upon cisplatin exposure compared to controls, underscoring the link between mitochondrial dysfunction, CSC properties, and chemoresistance. These cells also showed greater survival following cisplatin treatment compared to controls, reinforcing the link between mitochondrial dysfunction, stemness acquisition, and chemoresistance [25]. Kubo et al. further quantified chemoresistance in TFAM-silenced TE11 cells, demonstrating an increase in the half-maximal inhibitory concentration (IC_50_) for cisplatin, 5FU, and docetaxel. Annexin V/PI apoptosis assays showed a decrease in apoptotic fraction from 23% in controls to 6–10% following 48 h cisplatin (5 µM) treatment [43]. These data indicate that mitochondrial dysfunction–driven EMT confers survival advantages under chemotherapeutic stress.

### 3.4. Epigenetic Remodeling via DNMT Upregulation

**Figure 3:**
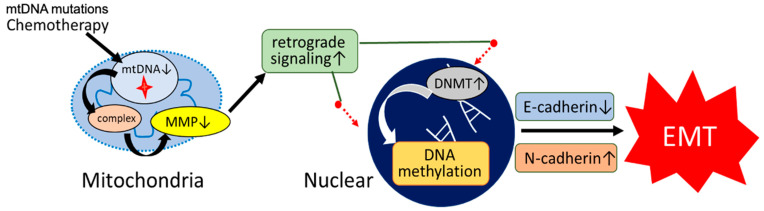
Proposed Model of mtDNA Dysfunction–Induced Epigenetic Reprogramming Leading to EMT and Chemoresistance. A conceptual model illustrating how mtDNA mutations may lead to mitochondrial dysfunction, triggering epigenetic reprogramming, epithelial–mesenchymal transition (EMT), and subsequent chemoresistance in ESCC. Chemotherapy and/or mtDNA mutations cause a reduction in mtDNA content, leading to impaired mitochondrial respiratory complex function and decreased mitochondrial membrane potential (MMP). This mitochondrial dysfunction triggers enhanced retrograde signaling from the mitochondria to the nucleus, which in turn upregulates DNA methyltransferase (DNMT) expression. Increased DNMT activity promotes aberrant DNA methylation of epithelial marker genes (e.g., *CDH1*), resulting in decreased E-cadherin and increased N-cadherin expression. These changes facilitate EMT, contributing to tumor progression and chemoresistance. Figure adapted from Kubo et al. (2022) [43].

Epigenetic alterations—chiefly DNA methylation—play pivotal roles in silencing tumor suppressor genes and modulating phenotypic plasticity [44]. DNMT family members (DNMT1, DNMT3A, DNMT3B) catalyze 5-methylcytosine formation at CpG dinucleotides, enforcing heritable gene repression. Recent findings in ESCC have elucidated a mitochondria-to-nucleus retrograde signaling axis linking mitochondrial dysfunction to DNMT-mediated epigenetic remodeling. Kubo et al. reported that loss of mitochondrial DNA (mtDNA) content leads to the depolarization of the mitochondrial membrane potential (Δψm), which subsequently upregulates DNMT1, DNMT3A, and DNMT3B expression at both the mRNA and protein levels. This upregulation increases global 5-methylcytosine content by approximately 10%, and promotes the decreased expression of key epithelial markers, such as CDH1 (E-cadherin), thereby enhancing epithelial–mesenchymal transition (EMT) and drug-resistant phenotypes. These epigenetic changes are accompanied by the increased expression of mesenchymal markers including N-cadherin, Vimentin, and ZEB1, and are functionally associated with heightened invasiveness and chemoresistance to agents such as cisplatin (CDDP), 5-fluorouracil (5-FU), and docetaxel. Pharmacological inhibition of DNMTs using zebularine reversed EMT features in mtDNA-depleted ESCC cells by demethylating the E-cadherin promoter, restoring its expression, and reducing levels of mesenchymal markers. Notably, zebularine treatment reduced 5-methylcytosine levels by approximately 10%, increased E-cadherin protein levels, and decreased the IC_50_ of cisplatin by 40% in these cells, indicating that epigenetic targeting can partially restore drug sensitivity under conditions of mitochondrial dysfunction (Figure 3) [43].

These results underscore the existence of a mitochondrial epigenetic circuit, wherein mtDNA depletion and mitochondrial membrane depolarization act as upstream triggers of DNMT activation, leading to stable, heritable gene repression that drives phenotypic reprogramming and chemoresistance. This Δψm-dependent modulation of nuclear gene expression highlights the intimate cross-talk between mitochondrial bioenergetics and chromatin structure, suggesting that metabolic cues can directly inform epigenetic states in cancer. The mechanistic foundation for this phenomenon is grounded in the concept of mitochondrial retrograde signaling, as first detailed by Butow and Avadhani. This evolutionarily conserved signaling pathway transmits functional states of mitochondria—such as changes in membrane potential, ROS levels, calcium flux, and metabolite availability—to the nucleus, thereby altering gene expression to compensate for mitochondrial stress. Retrograde signaling serves not merely as a stress response but also as an epigenetic rheostat that shapes chromatin landscapes in accordance with mitochondrial status [45].

Supporting this, Smiraglia et al. demonstrated that mtDNA-deficient (ρ0) cells exhibit the upregulation of DNMT1, DNMT3A, and DNMT3B, leading to increased global DNA methylation and the transcriptional silencing of key nuclear genes [46]. Importantly, these changes occurred independently of exogenous oxidative stress, suggesting that intrinsic mitochondrial dysfunction is sufficient to initiate epigenetic remodeling via retrograde signaling. Taken together, these findings position mitochondrial dysfunction not as a passive metabolic defect but as an active regulator of the nuclear epigenome. In the context of ESCC, where mitochondrial stress and epigenetic plasticity converge to drive EMT and therapeutic resistance, targeting the mitochondrial–epigenetic interface represents a rational and potentially effective therapeutic approach. Future studies should explore whether mitochondrial retrograde signaling also governs other chromatin-modifying systems—including TET enzymes, histone deacetylases (HDACs), or Polycomb group proteins—and how these axes integrate with oncogenic and inflammatory pathways (e.g., NF-κB, STAT3) to fine-tune the cancer cell phenotype.

## 4. Therapeutic Strategies Targeting Mitochondrial Vulnerabilities

Given the central role of mitochondrial dysfunction in ESCC chemoresistance, several therapeutic avenues have emerged that specifically target mitochondrial biology. Below, we discuss three major strategies: (1) DNMT inhibitors to reverse epigenetic reprogramming; (2) bioenergetic modulators to exploit metabolic dependencies; (3) mitochondrial genome-editing technologies to correct mtDNA mutations or eliminate mutant genomes.

### 4.1. Targeting Epigenetic Dysregulation with DNMT Inhibitors

Zebularine and decitabine, nucleoside analog DNMT inhibitors, have shown efficacy in preclinical EC models. Zebularine reversed EMT markers and chemoresistance in mtDNA-depleted cells. A combination with cisplatin improved apoptosis rates and decreased xenograft tumor volume [43]. Phase I trials of decitabine in solid tumors report tolerable safety profiles, suggesting feasibility for combination regimens [47].

### 4.2. Exploiting Metabolic Vulnerabilities with Bioenergetic Modulators

Biguanides (metformin, phenformin) inhibit Complex I, further impairing ATP production in cancer cells [48]. High-OXPHOS pancreatic cancers exhibit chemoresistance, which can be overcome by targeting mitochondrial Complex I with phenformin, sensitizing tumors to gemcitabine treatment [49]. Mitochondria-targeted antioxidants such as mitoquinone (MitoQ) can paradoxically increase mitochondrial ROS at higher concentrations [50]. Cheng et al. demonstrated that combined with the glycolysis inhibitor 2-deoxyglucose (2-DG), which blocks alternative ATP production, the dual treatment induced synergistic cell death by depleting energy sources and increasing oxidative stress [51].

### 4.3. Mitochondrial DNA Editing Technologies

Mitochondrial DNA (mtDNA) mutations contribute to a wide range of human diseases including mitochondrial disorders, aging, neurodegeneration, and cancers. Unlike nuclear DNA, editing of mtDNA has long remained a technical challenge due to the compartmentalization of mitochondria.

TALENs (transcription activator-like effector nucleases) can be customized to bind specific DNA sequences and have been adapted for mitochondrial use by fusing with mitochondrial targeting signals (MTS). Gammage et al. developed mitoTALENs capable of selectively eliminating mutant mtDNA without affecting the wild-type genome in human osteosarcoma 143B cybrid cell lines harboring pathogenic mutations [52]. This selective removal promoted the replication of wild-type mtDNA, effectively shifting heteroplasmy and restoring mitochondrial function, such as ATP production and membrane potential. While the study demonstrated improved bioenergetic parameters, it did not directly assess whether these changes conferred enhanced chemoresistance.

Recent advances in mitochondrial genome editing have been driven by the development of CRISPR-free base editors, such as DdCBEs (DddA-derived cytosine base editors), which enable targeted C-to-T conversions in mtDNA without the need for guide RNAs [53]. This system utilizes split DddAtox (a cytidine deaminase) fused to mitoTALENs, enabling site-specific base editing within the mitochondrial genome. DdCBE has since been used to generate disease models by replicating pathogenic mtDNA mutations. However, challenges remain, particularly regarding off-target effects and efficient delivery into mitochondria. To address this limitation, Lee et al. engineered high-fidelity DdCBEs (HiFi-DdCBEs) by introducing alanine substitutions at key interface residues of the DddAtox split domain [54]. This modification prevents deaminase reconstitution unless the TALE proteins bind adjacent mtDNA sites, thereby significantly reducing off-target activity.

Although CRISPR-Cas9 has revolutionized genome editing in nuclear DNA, its application to mtDNA has been hampered by the inability to transport gRNAs into mitochondria. However, recent studies have demonstrated potential workarounds. For example, Jo et al. (2015) engineered mitochondrially targeted Cas9 (mitoCas9) along with an imported gRNA, achieving partial editing of mtDNA in human cells [55]. These results suggest that while significant technical barriers remain, CRISPR-based mtDNA editing may become increasingly viable. Overall, these technologies represent a major step forward in the precise manipulation of the mitochondrial genome and hold promise for future therapeutic strategies.

## 5. Conclusions and Future Directions

Mitochondrial DNA alterations orchestrate a complex resistance network in EC, integrating bioenergetic reprogramming, ROS signaling, epigenetic remodeling, EMT/CSC induction, and intercellular mitochondrial dynamics. The comprehensive profiling of mitochondrial biomarkers such as mtDNA copy number or D-loop mutational burden, should guide patient stratification in clinical trials. Combination therapies targeting mitochondrial vulnerabilities, from DNMT inhibitors to gene-editing and bioenergetic modulators, promise to overcome chemoresistance. Future research must focus on translating these strategies into safe, effective clinical interventions and developing noninvasive diagnostics for the real-time monitoring of mitochondrial function during therapy.

## Data Availability

Not applicable.
